# Contemporary analysis of ETEST for antibiotic susceptibility and minimum inhibitory concentration agreement against *Pseudomonas aeruginosa* from patients with cystic fibrosis

**DOI:** 10.1186/s12941-021-00415-0

**Published:** 2021-01-19

**Authors:** Maxwell J. Lasko, Holly K. Huse, David P. Nicolau, Joseph L. Kuti

**Affiliations:** 1 Center for Anti-Infective Research and Development, Hartford Hospital, 80 Seymour Street, Hartford, 06102 CT USA; 2 Department of Clinical Microbiology, Huntington Hospital, Pasadena, CA USA; 3 Division of Infectious Diseases, Hartford Hospital, Hartford, CT USA; 4 Present Address: Department of Clinical Microbiology, Harbor-UCLA Medical Center, Torrance, CA USA

**Keywords:** Antibiotic resistance, Broth microdilution, Ceftazidime–avibactam, Ceftolozane–tazobactam

## Abstract

**Objectives:**

Cystic fibrosis (CF) acute pulmonary exacerbations are often caused by *Pseudomonas aeruginosa*, including multi-drug resistant strains. Optimal antibiotic therapy is required to return lung function and should be guided by in vitro susceptibility results. There are sparse data describing ETEST performance for CF isolates using contemporary isolates, methods and interpretation, as well as novel antibiotics, such as ceftazidime–avibactam and ceftolozane–tazobactam.

**Methods:**

*Pseudomonas aeruginosa* (n = 105) isolated during pulmonary exacerbation from patients with CF were acquired from 3 US hospitals. Minimum inhibitory concentrations (MICs) were assessed by reference broth microdilution (BMD) and ETEST for aztreonam, cefepime, ceftazidime, ceftazidime–avibactam, ceftolozane–tazobactam, ciprofloxacin, levofloxacin, meropenem, piperacillin–tazobactam, and tobramycin. Broth microdilution was conducted in concordance with the Clinical and Laboratory Standards Institute M100. ETEST methodology reflected package insert recommendations. Performance of ETEST strips was evaluated using the Food and Drug Administration (FDA) and Susceptibility Testing Manufacturers Association (STMA) guidance.

**Results:**

Of the 105 *P. aeruginosa* included, 46% had a mucoid phenotype. ETEST MICs typically read 0–1 dilution higher than BMD for all drugs. Categorical agreement and essential agreement ranged from 64 to 93% and 63 to 86%, respectively. The majority of observed errors were minor. A single very major error occurred with ceftazidime (4.2%). For ceftazidime–vibactam, 2 very major errors were observed and both were within essential agreement. Major errors occurred for aztreonam (3.3%), cefepime (9.4%), ceftazidime–avibactam (5.3%, adjusted 2.1%), ceftolozane–tazobactam (1%), meropenem (3.3%), piperacillin–tazobactam (2.9%), and tobramycin (1.5%).

**Conclusions:**

ETEST methods performed conservatively for most antibiotics against this challenging collection of *P. aeruginosa* from patients with CF.

## Introduction

Cystic fibrosis (CF) is the leading cause of mortality in Caucasians due to autosomal recessive disorders [1]. Once considered a disease with high pediatric mortality, innovations in therapy and care have improved the median age of survival to over 40 years [1–3]. These substantial survival improvements have largely been driven by advancements in prevention and treatment of CF acute pulmonary exacerbation, which is predominately caused by *Staphylococcus aureus* (early in the disease) and *Pseudomonas aeruginosa* (later in the disease) [3–5]. *P. aeruginosa* continues to be the most common identifiable bacterial pathogen causing chronic infection and acute pulmonary exacerbations, with upwards of 70% of CF patients infected with this Gram-negative organism as age approaches 40 years. Notably, ~ 20% of CF patients are infected with multi-drug-resistant (MDR) *P. aeruginosa*, likely due to numerous repetitive antibiotic courses received during treatment of acute pulmonary exacerbations [2–5].

Ideally, antibiotic selection during exacerbation is guided by culture and susceptibility data; furthermore, knowledge of the antibiotic minimum inhibitory concentration (MIC) enables additional optimization of antibiotic dosing regimens [6]. Automated antimicrobial susceptibility tests (AST) commonly used in the clinical microbiology lab perform poorly for CF isolates [7]. Therefore, the CF Foundation recommends the use of manual susceptibility test methods including broth microdilution (BMD) and agar based methods such as reference agar dilution, Kirby Bauer disc diffusion, and antibiotic gradient strips [8]. BMD and reference agar dilution are laborious and time-consuming for most clinical microbiology labs to consider. Additionally, Kirby Bauer disc diffusion only provides categorical susceptibility interpretation, and additional technology is required to translate disc zone sizes to MICs. Antibiotic gradient strips, such as ETEST^®^ (bioMérieux, Durham, NC), in contrast, are relatively simple to set up and provide specific MIC data for the tested antibiotic in 1–2 days. Previous studies demonstrated low error rates for ETEST against CF *P. aeruginosa*; however, the definitions used to assess performance are now outdated, and many of the tested antibiotics are no longer widely utilized by clinicians [9, 10]. Moreover, there is insufficient data describing the performance of ETEST for novel antibiotics such as ceftazidime–avibactam and ceftolozane–tazobactam against CF isolates. This study sought to evaluate ETEST performance using current guidance for 10 commonly utilized anti-pseudomonal antibiotics against a contemporary set of *P. aeruginosa* isolated from CF patients.

## Materials and methods

### Antibiotics

Analytical avibactam (AVI, Lot: 1192491614), aztreonam (ATM, Lot: MKBW2997V), cefepime (FEP, Lot: LRAB8503), ceftazidime (CAZ, Lot:117M4826V), ceftolozane (Lot: 44042000041), ciprofloxacin (CIP, Lot: 049M4087V), levofloxacin (LVX, Lot: BCCV4788), meropenem (MEM, Lot: LRAB7853), piperacillin (PIP, Lot:098M4886V), tazobactam (TAZ, Lot: 5/08), and tobramycin (TOB, Lot: 069M4782V) were purchased from Millipore Sigma (Burlington, MA) or MicroConstants (San Diego, CA). ETEST strips of each respective drug (ATM, Lot: 1007566240; FEP, Lot: 1007333550; CAZ, Lot: 1007837740; ceftazidime-avibactam [CZA], Lot: 1007754210; ceftolozane–azobactam [C/T], Lot: 1007622880; CIP, Lot: 1007821040; LVX, Lot: 1007663920; MEM, Lot: 9239528; piperacillin–tazobactam [TZP], Lot: 1007769270; and TOB, Lot: 1007750640) were provided by bioMérieux.

### *Pseudomonas aeruginosa* isolates

Non-duplicate clinical *P. aeruginosa* isolates were collected from patients during CF pulmonary exacerbation at United States CF centers in Hartford, Connecticut; Baltimore, Maryland; and Indianapolis, Indiana. Patient age at time of isolate collection and mucoid or non-mucoid morphology were recorded for each respective isolate; no protected health information was shared. Multiple isolates from individual patients were included if different mucoid morphology was recorded. Prior to experimentation, isolates were subcultured twice on trypticase soy agar with 5% sheep’s blood and allowed to incubate at 37 °C overnight. All isolates were frozen at − 80 °C until analysis.

### Minimum inhibitory concentration studies

All experiments were performed at the Center for Anti-Infective Research and Development (CAIRD) in triplicate. Prior to experimentation, 96 well microtiter MIC trays (Starstedt, Newton, NC) were prepared for each antibiotic with standard cation-adjusted Mueller–Hinton Broth (Lot: 9239528; Becton, Dickinson, and Company, Sparks, MD); trays were then subjected to quality control testing as outlined by the Clinical and Laboratory Standards Institute (CLSI) [11]. Antibiotic MICs were determined by BMD as referenced by CLSI [11]. Inoculums were prepared using a digital densitometer (Model Den-1B, GrantBio Inc. United Kingdom) and adjusted to achieve colony counts of 1–5 × 10^8^ CFU/ml as per instructions outlined in the ETEST package insert [12]. ETEST MIC testing was conducted from the same inoculum and run simultaneously with BMD for each antibiotic/isolate combination. Each inoculum was applied to a 150 mm Mueller–Hinton Agar plate (Lot: 0059390; Becton–Dickinson, Sparks, MD) with a sterile cotton swab and allowed to dry for 10 min. ETEST strips were then placed on inoculated plates using a Simplex C76 (bioMérieux, Durham, NC) device. BMD trays and agar plates were incubated for 18–20 h at 37 °C prior to reading the MIC. In rare scenarios, results that were not interpretable were re-incubated for up to a maximum of 48 h. Colony counts were performed for each inoculum prepared. If colony counts were outside the range, the MIC result was excluded, and both BMD and ETEST for the antibiotic/isolate combination were repeated with an adjusted McFarland. *P. aeruginosa* ATCC 27853 served as quality control on each experiment day. If the quality control did not meet its acceptable MIC or colony count range, all associated data were not recorded and replicates were repeated on a separate experiment day.

### Data analyses

All MICs were read by the naked eye and confirmed independently by two investigators at CAIRD. Discordance was arbitrated by a third reader. Modal MICs were used for all analyses. Reproducibility rates were recorded for all antibiotics. The 30th edition of CLSI M100 and 5th edition of M23 were utilized for MIC categorical interpretations and assessment of error rates, respectively [11, 13]. ETEST was considered within essential agreement (EA) if it was within 1 doubling dilution of the BMD MIC. Categorical agreement (CA) was defined as both reference and test methods in the same interpretative category. A minor error (miE) occurred when the reference method was interpreted as intermediate and the challenge test was interpreted as susceptible or resistant and vice versa. Major errors (ME) occurred if the reference method was interpreted susceptible and the challenge method was interpreted resistant. Conversely, a very major error (VME) was defined as the BMD being interpreted as resistant and ETEST susceptible. All observations of VME and ME were repeated to confirm results. Primary evaluation of acceptable error rates were evaluated using Food and Drug Administration (FDA) and Susceptibility Testing Manufacturers Association (STMA) guidance, which permits ME and VME within EA to be adjusted for drugs with no intermediate category (e.g., CZA) [14, 15]. miE within EA were also adjusted for all drugs and presented separately.

## Results

### *Pseudomonas aeruginosa* isolates

One-hundred and five *P. aeruginosa* isolates were available for analyses (Hartford, n = 35; Baltimore, n = 33; Indianapolis, n = 37). The median age of the patient with CF at isolate retrieval was 31 years, while 21 isolates (20%) were acquired from patients under the age of 18. A mucoid morphology was observed for 48 (46%). McFarland densities needed to achieve the required colony count (1–5 × 10^8^ CFU/ml) range were 0.7–1.0 for non-mucoid and 1.65–2.0 for mucoid *P. aeruginosa* isolates. The median incubation time until readable result was 20 h, while 13 (12.4%) and 3 (2.9%) isolates required 24 and 48 h of incubation, respectively.

### Minimum inhibitory concentration studies

Detailed MIC data and antibiotic susceptibility by reference BMD and ETEST are presented in Table 1. Forty-one *P. aeruginosa* (39%) were characterized as MDR, while ≥ 30% of isolates were distributed within 1 doubling dilution of the intermediate breakpoint for most of the antibiotics (exceptions: CZA, C/T, MEM). Median modal ETEST MICs read 0 or 1 dilution higher (IQR 0–1) than BMD for all tested antibiotics. There were nominal differences (≤ 5%) between susceptibility for non-mucoid and mucoid *P. aeruginosa* based on BMD results for all drugs with exception of CIP, LVX and MEM, as well as ETEST results with exception of CIP, LVX, MEM, TZP, and TOB (Additional file 1: Tables S1 and S2). Intra-isolate BMD MIC reproducibility spanned a 1 and 2 log dilution difference in 93% (IQR: 92–98) and 98% (IQR: 97–99) of occurrences across all antibiotics, respectively. Similarly, intra-isolate ETEST reproducibility covered a median 1 dilution in 96% (IQR: 95–99) and 2 dilution in 99% (IQR: 98–99) of isolates. Individual BMD and ETEST reproducibility results by antibiotic are provided in Additional file 1: Table S3.

**Table 1:** MIC_50_, MIC_90_, and percent susceptible, intermediate or resistant for 105 CF *P. aeruginosa* by reference broth microdilution and ETEST

Antibiotic	BMD			ETEST		
	MIC_50_ (µg/ml)	MIC_90_ (µg/ml)	S/I/R (%)	MIC_50_ (µg/ml)	MIC_90_ (µg/ml)	S/I/R (%)
ATM	8	64	58/10/32	8	≥ 256	54/11/35
FEP	8	≥ 128	50/20/30	16	≥ 256	32/21/47
CAZ	4	64	68/10/22	4	≥ 256	74/6/20
CZA	2/4	8/4	90/-/10	2/4	16/4	88/-/12
C/T	1/4	4/4	92/2/6	2/4	8/4	87/6/7
CIP	2	8	27/14/59	2	≥ 32	27/14/59
LVX	4	16	24/13/63	16	≥ 32	20/8/72
MEM	1	32	58/6/36	1	≥ 32	55/5/40
TZP	8/4	256/4	67/13/20	8/4	≥ 256/4	58/15/27
TOB	2	32	63/13/24	4	64	50/20/30

ATM: aztreonam; FEP: cefepime; CAZ: ceftazidime; CZA: ceftazidime–avibactam; C/T: ceftolozane–tazobactam; CIP: ciprofloxacin; LVX: levofloxacin; MEM: meropenem; TZP: piperacillin–tazobactam; TOB: tobramycin; S: susceptible; I: intermediate; R: resistant; MIC_50_: minimum inhibitory concentration of 50% of isolates; MIC_90_: minimum inhibitory concentration of 90% of isolates

### ETEST performance

The performance of the ETEST is provided in Table 2. EA and CA ranged from 63 to 86% and 64 to 93%, respectively. A single VME was observed with CAZ, causing the drug to be above the threshold. Two VMEs were observed with CZA; however, both errors were within EA and therefore adjusted for final interpretation. Unacceptable MEs were observed for ATM, FEP, and MEM. Five ME were observed for CZA but three were within EA and adjusted for final interpretation. The majority of errors for all antibiotics, except CZA, were miE (range: 5.7% for C/T to 31.4% for FEP), and EA was < 90% for all drugs tested. Consideration of EA on miE reduced this range to 1.0% for C/T to 12.4% for FEP. ETEST performance partitioned by mucoid versus non-mucoid morphology is provided in Additional file 1: Tables S4 and S5. Observed errors were evenly split between mucoid and non-mucoid morphologies.

**Table 2:** ETEST performance based on FDA guidance

Antibiotic	EA (%)	CA (%)	VME* No. (%)	ME* No. (%)	miE* No. (%)	Recalculated miE** No. (%)
ATM	78	87	0	2 (3.3%)	13 (12.4%)	5 (4.8%)
FEP	72	64	0	5 (9.4%)	33 (31.4%)	13 (12.4%)
CAZ	82	91	1 (4.2%)	0	9 (8.6%)	3 (2.9%)
CZA	78	93	2 (18.2%)/0 (0.0%)*	5 (5.3%)/2 (2.1%)*	ND	ND
C/T	86	93	0	1 (1.4%)	6 (5.7%)	1 (1.0%)
CIP	79	8	0	0	16 (15.2%)	5 (4.8%)
LVX	63	83	0	0	18 (17.1%)	8 (7.6%)
MEM	86	91	0	2 (3.3%)	7 (6.7%)	3 (2.9%)
TZP	77	85	0	2 (2.9%)	14 (13.3%)	11 (10.5%)
TOB	82	81	0	1 (1.5%)	19 (18.1%)	6 (5.7%)

EA: essential agreement; CA: categorical agreement; VME: very major error; ME: major error; miE: minor error; ND: not done

*FDA thresholds for VME, ≤ 2%; ME, < 3%; no criteria for miE; VME and ME thresholds based on adjusted analysis (excluding EA) for CZA due to absence of intermediate breakpoint

**Recalculated minor errors (excluding EA)

## Discussion

Acute pulmonary exacerbations in patients with CF are commonly caused by *P. aeruginosa*, and with repeated exposures to antibiotics, multidrug resistance is frequently observed [3, 4, 8, 16]. Most clinical laboratories employ agar based AST methods to test CF isolates, since BMD is impractical and automated systems have historically performed poorly for such isolates [7]. Gradient diffusion strips, including ETEST, are one such method recommended by the Cystic Fibrosis Foundation; however, contemporary data on their performance are lacking, particularly for newer antibiotics, such as CZA and C/T, that may play a role in treatment of MDR *P. aeruginosa* exacerbations [17–20]. In this study, ETEST performance was compared with reference BMD for 10 antibiotics commonly utilized during acute pulmonary exacerbation against a contemporary collection of CF *P. aeruginosa.*

All ETEST strips evaluated in this study are cleared by FDA 510 k guidance for *P. aeruginosa*. Specific to the newer agents, CZA and C/T, a number of studies have reported acceptable performance for ETEST relative to reference BMD [21–26]. Notably, none of these studies reported inclusion of CF isolates, which not only may have higher rates of antibiotic resistance, but also more likely to overexpress the alginate exopolysaccharide, develop biofilms, and have a number of unique growth characteristics and phenotypes [27, 28]. In our study, FDA thresholds for VME and ME were met by all antibiotic ETEST strips except ATM, CAZ, FEP, and MEM, though EA was < 90% for all drugs tested. With the exception of a high number of ME for FEP, the other VME or ME scenarios were classified above threshold by only 1 isolate. In contrast, most of the observed errors were miE. Rates of miE exceeded 10% for ATM, FEP, CIP, LVX, TZP, and TOB. These higher rates of miE are likely a result of isolate BMD distributions at or near the breakpoint for this resistant population. The natural 1–2 doubling dilution difference frequently observed in MIC methodologies becomes burdensome when nearly a third of isolates are distributed within 1 dilution of the intermediate break point. In fact, 79 (59%) of the observed miE were within essential agreement. Furthermore, ETEST reproducibility rates were high across all antibiotics, demonstrating intra-method reliability. Clinical microbiology labs will most likely only determine antibiotic MICs to a respective isolate in singlet and need to rely on methods that demonstrate excellent reproducibility.

Across all antibiotics, the median MIC determined by ETEST was the same or 1 dilution higher than BMD. However, there were some isolates for which the ETEST read ≥ 2 dilutions from BMD, thus resulting in EA rates that were lower than CA. As a result, none of the tested antibiotics achieved greater than 90% EA, and only CAZ, C/T, MEM and TOB achieved at least 80% EA. Despite less than optimal EA, the majority (66.7–91.3%) of scenarios for which the ETEST MIC was ≥ 2 dilutions from BMD were still within categorical agreement (i.e., both isolates defined as susceptible or both as resistant) (data not shown). FEP (37.9%) and TZP (45.8%), however, displayed lower categorical agreement for isolates without EA.

Differences in EA and CA between non-mucoid and mucoid isolates were nominal (< 10%) for most antibiotics, with the exception of LVX. This is supported by the observations that errors were numerically distributed evenly between both phenotypes (Additional file 1: Tables S4, S5). Differences in the denominator affect rates of error and might give a false notion that higher rates of error occur with different phenotypes. Since there was a relatively small number of isolates in each morphology cohort, additional studies comparing mucoid vs. non-mucoid phenotypes are recommended.

These observations are not unlike previous studies conducted over a decade ago assessing ETEST performance against CF *P. aeruginosa* isolates [10]. Burns and colleagues used a composite analysis to conclude that rates of very major error (0.1%) and major error (2.2%) for several antibiotic tested herein (ATM, CAZ, CIP, MEM, TZP, and TOB) were within acceptable range. It is important to note that methods in this study used a different calculation for ME and VME rates (number of respective errors/total number of tested isolates) than the current, more stringent and contemporary FDA and CLSI methods (number of respective errors/number of reference method susceptible [ME] or resistant [VME] isolates), which were used in our study calculation [13, 29]. Thus, a direct comparison of error rates would not be appropriate. However, when assessed by individual antibiotic, VMEs were observed for CAZ, TZP, and ATM in their study, and unacceptable MEs were reported for TZP and MEM. Like our data, the majority of the observed errors were miE, ranging from 11.5% for ATM to 24.7% for CIP.

Collectively, the trend towards more ME than VME and higher MIC reading by ETEST suggests that MICs and interpretation of susceptibility will be conservative. As a result, there is a low likelihood that ETEST results will contribute to poorer than expected outcomes. While potentially elevated MICs may be observed with the ETEST method, these values may also assist in optimizing antibiotic dosing regimens. Several case reports have reported success in treating CF acute pulmonary exacerbation caused by MDR *P. aeruginosa* using high-dose, pharmacodynamically optimized regimens based on ETEST derived MICs and therapeutic drug monitoring [19, 20, 30].

There are some limitations to be noted in our study. First, our study was relative small with 105 isolates, although this number is above guidelines provided by CLSI and the FDA. Nonetheless, given very high non-susceptibility for this population, the smaller denominators for susceptible and resistant populations during assessment of VME and ME rates may inflate the effect that even one isolate has with respect to crossing error thresholds. Our small numbers also made formal analysis of mucoid vs non-mucoid morphology challenging, although numerically, the isolates producing errors appeared to be evenly distributed between the phenotypes. Second, clinical laboratories should be aware that a higher inoculum density was required for these non-mucoid (0.7–1 McFarland) and mucoid (1.65–2 McFarland) CF *P. aeruginosa* isolates to achieve appropriate colony count ranges in the 1–5 × 10^8^ CFU/ml range. The ETEST package insert recommends McFarland adjustment to achieve the specified colony count range before interpretation of results. Finally, MIC testing was conducted at a single-center using frozen isolates. Conducting this experiment over multiple sites and using fresh clinical isolates would add robustness to this study and could be explored in future studies.

## Conclusions

In summary, while ETEST did not meet EA criteria (≥ 90%) for the drugs tested, most of the errors detected were minor errors, and these discrepancies would likely not affect antibiotic decisions. Unacceptable ME rates were observed for ATM, FEP, and MEM, while there was a single VME for CAZ. New agents CZA and C/T passed all criteria except EA. ETEST was reproducible and provided a conservative estimate of MIC that was most often similar or 1 dilution above the reference MIC. Clinical microbiology labs should be aware of VME, ME, and miE that may occur with ETEST for some antibiotics against CF *P. aeruginosa*, as well as the higher initial McFarland required to achieve appropriate colony count ranges for the test, as instructed in the ETEST package insert.

## Supplementary information

**Additional file 1.** Additional Tables.

## Data Availability

Data can be provided upon reasonable request to the corresponding author.

## Abbreviations

CF: Cystic fibrosis
MDR: Multi-drug-resistant
MIC: Minimum inhibitory concentration
AST: Automated susceptibility tests
BMD: Broth microdilution
AVI: Avibactam
ATM: Aztreonam
FEP: Cefepime
CAZ: Ceftazidime
CIP: Ciprofloxacin
LVX: Levofloxacin
MEM: Meropenem
PIP: Piperacillin
TAZ: Tazobactam
TOB: Tobramycin
CZA: Ceftazidime–avibactam
C/T: Ceftolozane–tazobactam
TZP: Piperacillin–tazobactam
CAIRD: Center for Anti-Infective Research and Development
CLSI: Clinical and Laboratory Standards Institute
EA: Essential agreement
CA: Categorical agreement
miE: Minor error
ME: Major error
VME: Very major error
FDA: Food and Drug Administration
STMA: Susceptibility Test Manufacturers Association

## References

[1] Sanders DB, Fink AK (2016). Background and epidemiology. Pediatr Clin North Am.

[2] Davis PB (2006). Cystic fibrosis since 1938. Am J Respir Crit Care Med.

[3] Elborn JS (2016). Cystic fibrosis. Lancet.

[4] Emerson J, McNamara S, Buccat AM, Worrell K, Burns JL (2010). Changes in cystic fibrosis sputum microbiology in the United States between 1995 and 2008. Pediatr Pulmonol.

[5] Saiman L, Siegel J (2003). Infection control recommendations for patients with cystic fibrosis: microbiology, important pathogens, and infection control practices to prevent patient-to-patient transmission. Am J Infect Control.

[6] Kuti JL, Pettit RS, Neu N, Cies JJ, Lapin C, Muhlebach MS, Novak KJ, Nguyen ST, Saiman L, Nicolau DP (2018). Meropenem time above the MIC exposure is predictive of response in cystic fibrosis children with acute pulmonary exacerbations. Diagn Microb Infect Dis.

[7] Burns JL, Saiman L, Whittier S, Krzewinski J, Liu Z, Larone D, Marshall SA, Jones RN (2001). Comparison of two commercial systems (Vitek and MicroScan-WalkAway) for antimicrobial susceptibility testing of *Pseudomonas aeruginosa* isolates from cystic fibrosis patients. Diagn Microbiol Infect Dis.

[8] Cystic Fibrosis Foundation: Patient Registry Annual Data Report https://www.cff.org/Research/Researcher-Resources/Patient-Registry/2018-Patient-Registry-Annual-Data-Report.pdf. Published 2019.

[9] Balke B, Hogardt M, Schmoldt S, Hoy L, Weissbrodt H, Häussler S (2006). Evaluation of the E test for the assessment of synergy of antibiotic combinations against multiresistant *Pseudomonas aeruginosa* isolates from cystic fibrosis patients. Eur J Clin Microbiol Infect Dis.

[10] Burns JL, Saiman L, Whittier S, Larone D, Krzewinski J, Liu Z, Marshall SA, Jones RN (2000). Comparison of Agar diffusion methodologies for antimicrobial susceptibility testing of *Pseudomonas aeruginosa* isolates from cystic fibrosis patients. J Clin Microbiol.

[11] Clinical and Laboratory Standards Institute (CLSI) (2020). Performance Standards for Antimicrobial Susceptibility Testing.

[12] ETEST ceftazidime-avibactam. Package insert. bioMérieux Inc; 2017.

[13] Clinical and Laboratory Standards Institute (CLSI) (2018). Development of in vitro susceptibility testing criteria and quality control parameters. CLSI guideline M23.

[14] Federal Drug Administration (FDA). Guidance for Industry and FDA Class II Special Controls Guidance Document: Antimicrobial Suceptibility Test (AST) Sytems. Published March 5th 2007. Updated August 28, 2009. https://www.fda.gov/media/88069/download. Accessed 30 June 2020

[15] Kircher SM, Cullen SK, Killian S, Zimmer BL, Brasso BB (2016). The Susceptibility Testing Manufacturers Association presents an opinion for the delay of current susceptibility tests. Clin Infect Dis.

[16] Stefani S, Campana S, Cariani L, Carnovale V, Colombo C, Lleo MM, Iula VD, Minicucci L, Morelli P, Pizzamiglio G (2017). Relevance of multidrug-resistant *Pseudomonas aeruginosa* infections in cystic fibrosis. Int J Med Microbiol.

[17] Bensman TJ, Wang J, Jayne J, Fukushima L, Rao AP, D’Argenio DZ, Beringer PM (2017). Pharmacokinetic-pharmacodynamic target attainment analyses to determine optimal dosing of ceftazidime–avibactam for the treatment of acute pulmonary exacerbations in patients with cystic fibrosis. Antimicrob Agents Chemother.

[18] Monogue ML, Pettit RS, Muhlebach M, Cies JJ, Nicolau DP, Kuti JL (2016). Population pharmacokinetics and safety of ceftolozane–tazobactam in adult cystic fibrosis patients admitted with acute pulmonary exacerbation. Antimicrob Agents Chemother.

[19] Spoletini G, Etherington C, Shaw N, Clifton IJ, Denton M, Whitaker P, Peckham DG (2019). Use of ceftazidime/avibactam for the treatment of MDR *Pseudomonas aeruginosa* and *Burkholderia cepacia* complex infections in cystic fibrosis: a case series. J Antimicrob Chemother.

[20] Stokem K, Zuckerman JB, Nicolau DP, Wungwattana M, Sears EH (2018). Use of ceftolozane–tazobactam in a cystic fibrosis patient with multidrug-resistant *Pseudomonas* infection and renal insufficiency. Respir Med Case Rep.

[21] Bailey AL, Armstrong T, Dwivedi HP, Denys GA, Hindler J, Campeau S, Traczewski M, Humphries R, Burnham CA (2018). Multicenter evaluation of the Etest gradient diffusion method for ceftolozane–tazobactam susceptibility testing of *Enterobacteriaceae* and *Pseudomonas aeruginosa*. J Clin Microbiol.

[22] Humphries RM, Hindler JA, Magnano P, Wong-Beringer A, Tibbetts R, Miller SA (2018). Performance of ceftolozane-tazobactam Etest, MIC test strips, and disk diffusion compared to reference broth microdilution for β-lactam-resistant *Pseudomonas aeruginosa* isolates. J Clin Microbiol.

[23] Kresken M, Körber-Irrgang B (2018). Performance of the Etest for susceptibility testing of Enterobacterales (*Enterobacteriaceae*) and *Pseudomonas aeruginosa* toward ceftazidime-avibactam. J Clin Microbiol.

[24] Schaumburg F, Bletz S, Mellmann A, Becker K, Idelevich EA (2019). Comparison of methods to analyse susceptibility of German MDR/XDR *Pseudomonas aeruginosa* to ceftazidime/avibactam. Int J Antimicrob Agents.

[25] Shields RK, Clancy CJ, Pasculle AW, Press EG, Haidar G, Hao B, Chen L, Kreiswirth BN, Nguyen MH (2018). Verification of ceftazidime-avibactam and ceftolozane-tazobactam susceptibility testing methods against carbapenem-resistant Enterobacteriaceae and *Pseudomonas aeruginosa*. J Clin Microbiol.

[26] Wang Q, Zhang F, Wang Z, Chen H, Wang X, Zhang Y, Li S, Wang H (2020). Evaluation of the Etest and disk diffusion method for detection of the activity of ceftazidime-avibactam against Enterobacterales and *Pseudomonas aeruginosa* in China. BMC Microbiol.

[27] Mayer-Hamblett N, Ramsey BW, Kulasekara HD, Wolter DJ, Houston LS, Pope CE, Kulasekara BR, Armbruster CR, Burns JL, Retsch-Bogart G (2014). *Pseudomonas aeruginosa* phenotypes associated with eradication failure in children with cystic fibrosis. Clin Infect Dis.

[28] Mayer-Hamblett N, Rosenfeld M, Gibson RL, Ramsey BW, Kulasekara HD, Retsch-Bogart GZ, Morgan W, Wolter DJ, Pope CE, Houston LS (2014). *Pseudomonas aeruginosa* in vitro phenotypes distinguish cystic fibrosis infection stages and outcomes. Am J Respir Crit Care Med.

[29] Humphries RM, Ambler J, Mitchell SL, Castanheira M, Dingle T, Hindler JA, Koeth L, Sei K (2018). CLSI Methods Development and Standardization Working Group best practices for evaluation of antimicrobial susceptibility tests. J Clin Microbiol.

[30] Romano MT, Premraj S, Bray JM, Murillo LC (2020). Ceftolozane/tazobactam for pulmonary exacerbation in a 63-year-old cystic fibrosis patient with renal insufficiency and an elevated MIC to *Pseudomonas aeruginosa*. IDCases.

