# Supplementary material for: Contemporary analysis of ETEST for antibiotic susceptibility and minimum inhibitory concentration agreement against Pseudomonas aeruginosa from patients with cystic fibrosis
Source: Ann Clin Microbiol Antimicrob. 2021 Jan 19;20:9. doi: 10.1186/s12941-021-00415-0 (PMC7816365; doi:10.1186/s12941-021-00415-0)
Supplement: Supplementary file 1 — Additional file 1. Additional Tables. [file 12941_2021_415_MOESM1_ESM.pdf]

Evaluation of Etest for Antibiotic Susceptibility and Minimum Inhibitory Concentration Agreement against *Pseudomonas aeruginosa* from Patients with Cystic Fibrosis

Supplemental Material

Table S1. Broth microdilution generated susceptibility, MIC_{50} and MIC_{90} for each antibiotic against 105 CF *P. aeruginosa* by non-mucoid versus mucoid morphology

Antibiotic	%S		MIC_{50}		MIC_{90}	
	Non-Mucoid (n=57)	Mucoid (n=48)	Non-Mucoid (n=57)	Mucoid (n=48)	Non-Mucoid (n=57)	Mucoid (n=48)
ATM	60	56	8	8	64	64
FEP	51	50	8	8	≥128	≥128
CAZ	68	67	4	4	64	64
CZA	91	88	2/4	1/4	8/4	16/4
CT	93	92	1/4	1/4	4/4	4/4
CIP	33	21	2	2	8	4
LVX	28	19	4	4	16	16
MEM	65	50	0.5	2	32	32
TZP	68	65	8/4	8/4	256/4	256/4
TOB	65	60	4	2	32	16

ATM, aztreonam; FEP, cefepime; CAZ, ceftazidime; CZA, ceftazidime-avibactam; CT, ceftolozane-tazobactam; CIP, ciprofloxacin; LVX, levofloxacin; MEM, meropenem; TZP, piperacillin-tazobactam; TOB, tobramycin; S, susceptible; MIC_{50}, minimum inhibitory concentration of 50% of isolates; MIC_{90}, minimum inhibitory concentration of 90% of isolates

Table S2. Etest generated susceptibility, MIC_{50} and MIC_{90} for each antibiotic against 105 CF *P. aeruginosa* by non-mucoid versus mucoid morphology

Antibiotic	%S		MIC_{50}		MIC_{90}	
	Non-Mucoid (n=57)	Mucoid (n=48)	Non-Mucoid (n=57)	Mucoid (n=48)	Non-Mucoid (n=57)	Mucoid (n=48)
ATM	56	52	8	8	≥256	≥256
FEP	32	31	16	32	≥256	≥256
CAZ	72	77	2	4	≥256	128
CZA	88	85	4/4	2/4	16/4	16/4
CT	88	88	2/4	2/4	8/4	8/4
CIP	30	23	4	2	≥32	≥32
LVX	25	15	16	16	≥32	≥32
MEM	61	48	1	4	≥32	≥32
TZP	63	52	8/4	16/4	≥256/4	≥256/4
TOB	47	54	8	4	64	32

Table S3. Broth microdilution and Etest intra-isolate reproducibility results for individual antibiotics

Drug	BMD		Etest	
	Within 1 log	Within 2 log	Within 1 log	Within 2 log
ATM	92%	95%	96%	99%
FEP	92%	98%	100%	100%
CAZ	91%	95%	95%	99%
CZA	93%	98%	97%	97%
CT	92%	98%	99%	99%
CIP	93%	99%	95%	99%
LVX	95%	99%	95%	99%
MEM	95%	99%	91%	98%
TZP	93%	96%	91%	96%
TOB	97%	99%	99%	100%

BMD, broth microdilution

Table S4. Etest performance based on FDA criteria for 57 CF *P. aeruginosa* with non-mucoid morphology

Antibiotic	EA (%)	CA (%)	VME* (%)	ME* (%)	miE* (%)	Adjusted miE** (%)
ATM	79%	86%	0	1 (2.9%)	7 (12.3%)	3 (5.3%)
FEP	70%	68%	0	2 (6.9%)	16 (28.1%)	4 (7.0%)
CAZ	83%	93%	0	0	4 (7.0%)	2 (3.5%)
CZA	81%	97%	0<br>0	2 (3.8%)<br>0 (0.0%**)	NA	NA
CT	86%	93%	0	0	4 (7.0%)	0 (0.0%)
CIP	83%	90%	0	0	6 (10.5%)	2 (3.5%)
LVX	70%	81%	0	0	11 (19.3%)	4 (7.0%)
MEM	84%	95%	0	1 (2.7%)	2 (3.5%)	1 (1.8%)
TZP	79%	90%	0	1 (2.6%)	5 (8.8%)	5 (8.8%)
TOB	79%	77%	0	1 (2.7%)	11 (19.3%)	5 (8.8%)

EA, essential agreement; CA, categorical agreement; VME, very major error; ME, major error; miE, minor error; NA, not applicable

* FDA threshold for VME, ≤2%; ME, <3%; no criteria for miE

** Adjusted errors (excluding errors within EA) applied to miE for all antibiotics and VME/ME for drugs without 'Intermediate' category

Table S5. Etest performance based on FDA criteria for 48 CF *P. aeruginosa* with mucoid morphology

**Antibiotic**	**EA (%)**	**CA (%)**	**VME* (%)**	**ME* (%)**	**miE* (%)**	**Adjusted miE** (%)**
ATM	77%	88%	0	1 (3.7%)	5 (10.4%)	2 (4.2%)
FEP	75%	58%	0	3 (12.5%)	17 (35.4%)	7 (14.7%)
CAZ	81%	88%	1 (11.1%)	0	5 (10.4%)	1 (2.1%)
CZA	75%	90%	2 (33.3%)<br>0 (0.0%)**	3 (7.1%)<br>2 (4.2%)**	ND	ND
CT	85%	94%	0	1 (2.3%)	2 (4.2%)	1 (2.1%)
CIP	75%	79%	0	0	10 (20.8%)	3 (6.3%)
LVX	54%	85%	0	0	7 (14.6%)	4 (8.3%)
MEM	88%	88%	0	1 (4.2%)	5 (10.4%)	2 (4.2%)
TZP	75%	79%	0	1 (3.2%)	9 (18.8%)	(12.5%)
TOB	85%	85%	0	0	7 (14.7%)	1 (2.1%)

* FDA threshold for VME, ≤2%; ME, <3%; no criteria for miE

** Adjusted errors (excluding errors within EA) applied to miE for all antibiotics, and VME/ME for drugs without ‘Intermediate’ category

Table S6. Etest performance based on CLSI error rate bound method for 57 CF *P. aeruginosa* with non-mucoid morphology

Antibiotic		Count	miE	ME	VME
	≥I + 2	13	7.7%	ND	0.0%
ATM	I+ 1 to I-1	20	30.0%	5.0%	0.0%
	≤ I - 2	24	0.0%	0.0%	ND
	≥I + 2	9	11.1%	ND	0.0%
FEP	I+ 1 to I-1	27	44.4%	3.7%	0.0%
	≤ I - 2	21	14.3%	4.8%	ND
	≥I + 2	9	0.0%	ND	0.0%
CAZ	I+ 1 to I-1	16	25.0%	0.0%	0.0%
	≤ I - 2	32	0.0%	0.0%	ND
	R+1	1	-	ND	0.0%
CZA	R+S	11	-	18.2%	0.0%
	S-1	45	-	0.0%	ND
	≥I + 2	1	0.0%	ND	0.0%
CT	I+ 1 to I-1	10	40.0%	0.0%	0.0%
	≤ I - 2	46	0.0%	0.0%	ND
	≥I + 2	20	0.0%	ND	0.0%
CIP	I+ 1 to I-1	23	21.7%	0.0%	0.0%
	≤ I - 2	14	7.1%	0.0%	ND
	≥I + 2	27	0.0%	ND	0.0%
LVX	I+ 1 to I-1	17	64.7%	0.0%	0.0%
	≤ I - 2	13	0.0%	0.0%	ND
	≥I + 2	15	0.0%	ND	0.0%
MEM	I+ 1 to I-1	8	25.0%	12.5%	0.0%
	≤ I - 2	34	0.0%	0.0%	ND
	≥I + 2	8	0.0%	ND	0.0%
TZP	I+ 1 to I-1	13	30.8%	0.0%	0.0%
	≤ I - 2	36	2.8%	2.8%	ND
	≥I + 2	12	0.0%	ND	0.0%

TOB	I+ 1 to I-1	17	47.1%	5.8%	0.0%
	≤ I - 2	28	14.3%	0.0%	ND
CLSI m23 Criteria	≥I + 2 or ≥R+1		<5%	ND	<2%
	I+1 to I-1 or R+S		<40%	<10%	<10%
	≤ I - 2 or ≤ S - 1		<5%	<2%	ND

≥I + 2, MIC greater than or equal to 2 doubling dilutions than intermediate breakpoint; I+1 to I-1, MIC within 1 doubling dilution of intermediate breakpoint; ≤ I – 2, MIC less than or equal to 2 doubling dilutions lower than intermediate breakpoint; ≥R+1, MIC greater than or equal to 1 doubling dilution greater than resistant breakpoint; R+S, MIC at resistant or susceptible breakpoint; ≤ S – 1, MIC less than or equal to 1 doubling dilution lower than susceptible breakpoint

Table S7. Etest performance based on the CLSI error rate bound method for 48 CF *P. aeruginosa* with mucoid morphology

Antibiotic		Count	miE	ME	VME
	≥I + 2	10	0.0%	ND	0.0%
ATM	I+ 1 to I-1	15	26.7%	6.7%	0.0%
	≤ I - 2	23	4.4%	0.0%	ND
	≥I + 2	9	0.0%	ND	0.0%
FEP	I+ 1 to I-1	22	59.1%	9.1%	0.0%
	≤ I - 2	17	23.5%	5.9%	ND
	≥I + 2	7	0.0%	ND	14.3%
CAZ	I+ 1 to I-1	13	38.5%	0.0%	0.0%
	≤ I - 2	28	0.0%	0.0%	ND
	R+1	2	-	ND	0.0%
CZA	R+S	6	-	16.7%	33.3%
	S-1	40	-	5.0%	ND
	≥I + 2	2	0.0%	ND	0.0%
CT	I+ 1 to I-1	8	25.0%	0.0%	0.0%
	≤ I - 2	38	0.0%	2.6%	ND
	≥I + 2	16	6.3%	ND	0.0%
CIP	I+ 1 to I-1	23	34.8%	0.0%	0.0%
	≤ I - 2	7	14.3%	0.0%	ND
	≥I + 2	22	4.6%	ND	0.0%
LVX	I+ 1 to I-1	18	27.8%	0.0%	0.0%
	≤ I - 2	8	12.5%	0.0%	ND
	≥I + 2	16	0.0%	ND	0.0%
MEM	I+ 1 to I-1	10	50.0%	0.0%	0.0%
	≤ I - 2	22	0.0%	4.6%	ND
	≥I + 2	6	16.7%	ND	0.0%
TZP	I+ 1 to I-1	18	27.8%	0.0%	0.0%
	≤ I - 2	24	12.5%	4.2%	ND
	≥I + 2	5	0.0%	ND	0.0%

TOB	I+1 to I-1	16	37.5%	0.0%	0.0%
	≤ I-2	27	3.7%	0.0%	ND
	≥ I+2 or ≥ R+1		<5%	ND	<2%
CLSI m23 Criteria	I+1 to I-1 or R+S		<40%	<10%	<10%
	≤ I-2 or ≤ S-1		<5%	<2%	ND
